# Gene Expression Profiling of Two Distinct Neuronal Populations in the Rodent Spinal Cord

**DOI:** 10.1371/journal.pone.0003415

**Published:** 2008-10-15

**Authors:** Jesper Ryge, Ann-Charlotte Westerdahl, Preben Alstrøm, Ole Kiehn

**Affiliations:** 1 Mammalian Locomotor Laboratory, Department of Neuroscience, Karolinska Institutet, Stockholm, Sweden; 2 CORE A/S, Frederiksberg, Denmark; Emory University, United States of America

## Abstract

**Background:**

In the field of neuroscience microarray gene expression profiles on anatomically defined brain structures are being used increasingly to study both normal brain functions as well as pathological states. Fluorescent tracing techniques in brain tissue that identifies distinct neuronal populations can in combination with global gene expression profiling potentially increase the resolution and specificity of such studies to shed new light on neuronal functions at the cellular level.

**Methodology/Principal Findings:**

We examine the microarray gene expression profiles of two distinct neuronal populations in the spinal cord of the neonatal rat, the principal motor neurons and specific interneurons involved in motor control. The gene expression profiles of the respective cell populations were obtained from amplified mRNA originating from 50–250 fluorescently identified and laser microdissected cells. In the data analysis we combine a new microarray normalization procedure with a conglomerate measure of significant differential gene expression. Using our methodology we find 32 genes to be more expressed in the interneurons compared to the motor neurons that all except one have not previously been associated with this neuronal population. As a validation of our method we find 17 genes to be more expressed in the motor neurons than in the interneurons and of these only one had not previously been described in this population.

**Conclusions/Significance:**

We provide an optimized experimental protocol that allows isolation of gene transcripts from fluorescent retrogradely labeled cell populations in fresh tissue, which can be used to generate amplified aRNA for microarray hybridization from as few as 50 laser microdissected cells. Using this optimized experimental protocol in combination with our microarray analysis methodology we find 49 differentially expressed genes between the motor neurons and the interneurons that reflect the functional differences between these two cell populations in generating and transmitting the motor output in the rodent spinal cord.

## Introduction

The microarray technology combined with laser microdissection (LMD) makes it possible to study the gene expression profiles of identified cell populations [1], [2]. These advances have been embraced by the field of neuroscience to use the microarray expression profiles either as static classifiers of neuronal cell types in combination with more traditional anatomical and electrophysiological classification schemes [3]–[6] or to address the dynamics of global gene expression regulation within identified cell populations during development or in connection with disease and injury states [7], [8].

In the present study we aimed at establishing an experimental protocol that enabled us to compare the static gene expression profiles of fluorescently identified neuronal populations in the mammalian spinal cord that are directly involved in controlling and generating basic motor behaviors, like locomotion. We sampled neurons from the isolated rodent spinal cord of newborn animals, the dominant experimental model for the study of spinal networks that generate locomotion in mammals [9]–[12]. Two cell populations in the lumbar spinal cord that can be readily identified by fluorescent retrograde tracing [13] and which have been subject to extensive anatomical and electrophysiological characterization were examined here: the motor neurons (MNs) and the descending commissural interneurons (dCINs). These two groups of cells have distinct physiological functions in the spinal cord. The dCINs are integral elements of spinal interneuron networks that generate rhythmic locomotor movements and participate in the left-right coordination of hind limbs during locomotion [9], [14]–[17]. The MNs are principal output neurons of the spinal cord that transmit all motor related patterned activity to the muscles. Though being functionally distinct neuronal groups it is not know to what an extent these cell populations can be distinguished at the transcriptional level. However, differences in gene expression between neuronal cell types are likely to be relatively small, so both the experimental protocol and the subsequent analysis had to be optimized to detect small, but consistent, differences in gene expression. As part of this study we also introduce a new pre-processing method of microarray probe set intensity values that helps with the inspection of microarray quality and aides the choice of background compensation and normalization procedure. To identify differentially expressed (DE) genes we use a conglomerate classifier that for a given FDR threshold combines three existing methods, limma [18], Cyber-T [19] and SAM [20], to produce a set of significantly DE genes.

Our results show that amplified antisense RNA (aRNA) hybridized onto GeneChip® Rat Neurobiology U34 Arrays (RN_U34 chips) originating from as few as 50–250 cells detect 49 genes out of the 1050 annotated probe sets on these arrays as consistently differentially expressed between MNs and dCINs. In the MN population 17 genes were more expressed than in the dCIN population, while 32 genes were found to be more expressed in the dCIN population compared to the MNs. The DE genes reflect the anatomical and functional differences between these two neuronal populations. Together our results provide new insight to the transcriptional profiles of spinal neurons and outline an experimental approach that can be applied to examine the gene expression of individual cell populations in both the normal or diseased spinal cord.

## Results

### Cell identification, laser microdissection, isolation and amplification of RNA

**Figure 1:**
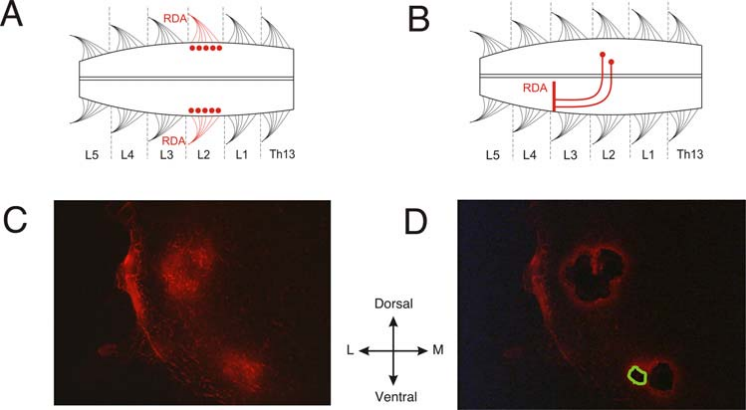
Labeling and laser microdissection. A and B show the schematic labeling procedure of cell populations in the lumbar spinal cord of the neonatal rat. RDA is applied to cut axons and retrogradely transported to the cell bodies. A shows MN labeling and B shows dCIN labeling. C and D illustrate the LMD of MNs in 10 µm thick spinal cord transections. Orientation: midline (M) is towards the right, lateral (L) is towards the left and ventral is downwards. C shows a spinal cord transection before LMD and D after LMD. The green thick outline illustrates vaporized tissue due to the laser action from the dissection of one cell, so in essence less tissue is dissected than the wholes might suggest. Each labeled cell is cut out separately to minimize contamination from surrounding cells.

In order to identify the two cell populations, MNs or dCINs, they were fluorescently labeled with the retrograde tracer rhodamine [dextran, tetramethylrhodamine (RDA)]. RDA labeling of MNs and dCINs is a well-established method in the rodent spinal cord [13], 21. As shown in Figures 1A and B, the RDA tracer is applied to cut axons of the target cells and retrogradely transported back to the cell bodies. After the RDA incubation we 1) immediately snap-froze the spinal cord with CO_2_, 2) cryo sectioned the lumbar (L) segment 2 into 10 µm sections which were subsequently mounted on polyester (POL) membrane slides and 3) laser microdissected the identified cells. The laser microdissected cells were lysed and the RNA was isolated, amplified and biotinylated giving good quality aRNA for microarray hybridization. An example of the LMD can be seen on Figures 1C and D.

### Microarray expression profiles and signal quality

Twenty-two microarrays were each hybridized with 5 µg amplified and biotinylated aRNA obtained from 50–250 laser microdissected cells originating from 22 separate animals, producing the following set of sample arrays: 7 MNs, 7 dCINs and 8 MIX (random sampling in the ventral horn). Before calculating gene expression summaries and exploring the data set for DE transcripts, the microarrays were pre-processed in order to transform all intensity values onto a common scale, i.e. background subtraction and normalization. Three background measures were tested: MAS, RMA and Global. Inspection of the probe intensity distributions after MAS or RMA background subtraction showed an extra peak at low intensities that were not present in the raw probe intensity distributions. To maintain the signal structure of the low intensity values and prevent the appearance of such a peak for low intensity probes, each array distribution was set to an intensity floor of zero by implementing the Global background subtraction.

**Figure 2:**
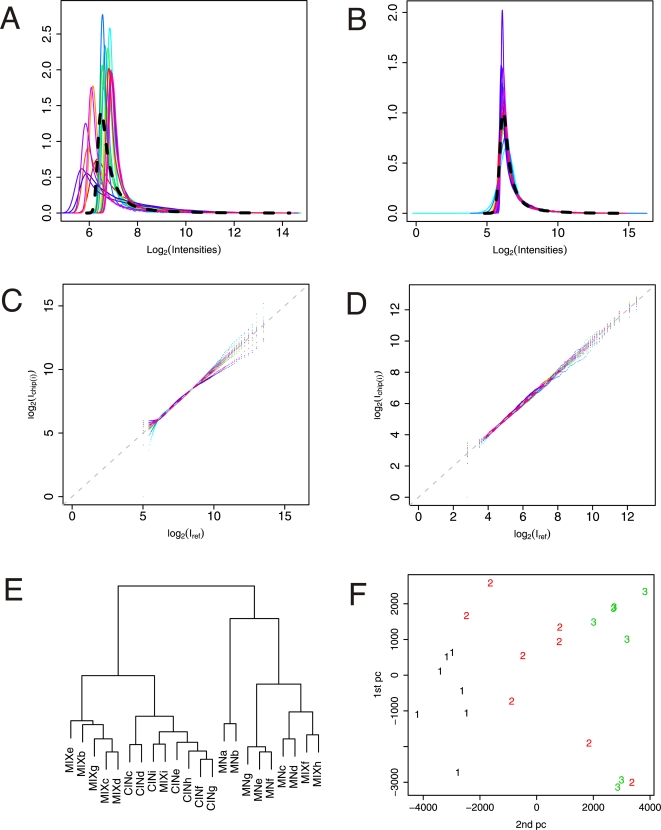
Affymetrix array normalization and quality control. The color code for each distribution is the same throughout A–D. A. The distributions of log_2_ transformed raw PM intensity values. The black dashed line illustrates the average distribution, *I_ref_*, of all 22 arrays (7 MNs, 7 dCINs and 8 MIX) in both A and B. B. QLT distributions of array PM intensities. C. qq-plots of the QLT distributions versus the average distribution. Deviations from *I_ref_* most pronounced at the distribution tails, i.e. at low and high intensities. D. qq-plot of Li-Wong summaries based on QLT+QPN PM values. E. Dendogram based on hierarchical clustering of correlation distance between individual microarray expression profiles (Li-Wong expression measures). The two major branches contain the two cell groups, MNs and dCINs respectively. MIX is dispersed in between the two groups. F. First two principal components of the gene expression profiles illustrating the Euclidian separation of individual microarrays. MNs and dCINs are nicely separated by the MIX, which are scattered in between these two groups. Symbols: 1 (black) = dCINs, 2 (red) = MIX, 3 (green) = MNs.

Following the Global background compensation we implement the quantile linear transformation (QLT) normalization procedure (see Materials and Methods). QLT is a linear transformation procedure we developed in order to visualize the generally accepted hypothesis that all samples have identical gene expression distributions, the principle of which is illustrated in Figure 2. Figure 2A shows the raw perfect match (PM) distributions of all 22 arrays plotted together with the average distribution. From these plots it is not apparent if all the raw distributions belong to a common expression distribution (e.g. the average distribution highlighted as the black dotted line) or if they constitute a set of different families of distributions (e.g. one distribution per experimental group). However, when the QLT distributions are plotted (Figure 2B) the clear superimpositions of these scaled curves do indeed indicate that all arrays in our study follow a common PM intensity distribution. When inspecting the quantile-quantile-plots (qq-plots) in Figure 2C we find small deviations in the distribution tails, which are less obvious in Figure 2B. These deviations are rather small and constitute less than 5% of the genes for each array. We assume that these nonlinear effects that the QLT distributions reveal are of experimental nature and not biological.

Expression summaries were calculated based on the normalized PM intensity values. QLT could have sufficed as a normalization procedure in its own right, but dendrograms (i.e. tree diagrams) based on correlation distances between gene expression profiles indicated that the addition of quantile projection normalization (QPN) [22] after QLT had a slight positive effect on the clustering of expression profiles into the correct experimental groups. Thus, RMA expression profiles based on either QLT or QPN alone failed to classify all arrays correctly according to experimental groups, whereas Li-Wong based on either normalization procedure did so correctly (not shown). However, both Li-Wong and RMA expression profiles based on QLT followed by QPN classified all the arrays correctly. This is illustrated for Li-Wong expression profiles in Figures 2E and F (and for RMA in Supplementary Figure S1). The dendrogram in Figure 2E shows that the arrays fall into two groups, dCINs and MNs, nicely classifying each array according to its biological cell type. The MIX population arrays intermingle with both these groups, which is expected since these arrays represent randomly laser microdissected cells including both dCINs and MNs. The separation of the experimental groups can also be seen in Figure 2F, where the first two principal components are shown for all array expression profiles. The separation of the arrays into the correct experimental groups based on the gene expression profiles indicates that the pattern of gene expression is indeed different between these two cellular groups and that it is captured in the array expression profiles. The fact that QLT followed by QPN seems to perform better than either QLT or QPN alone suggest that it is advantageous to separate the normalization into two steps that independently deals with the linear and nonlinear variations that may be present in the data set under examination. QLT handle linear experimental variations by rescaling all array distributions onto a common average distribution with a linear transformation, revealing any remaining nonlinear experimental variations as deviations of the QLT distributions from the average reference distribution. Significant nonlinear variations can subsequently be removed by a nonlinear transformation such as QPN.

As both QLT and QPN are performed at the probe level, we lastly examine if the PM distributional correlations are preserved in the expression summaries. Figure 2D shows the qq-plot of Li-Wong summary measures (after Global background subtraction, QLT and QPN), where the reference distribution again is the average quantile distribution this time calculated from the Li-Wong summary measures. This figure shows very good correlation among expression distributions verifying that the correlation at the probe level is indeed preserved after expression summary calculation. The same is true for RMA summaries (not shown). We therefore conclude that the QLT followed by QPN probe level normalization suffices and no additional normalization was carried out on the expression summaries. We use the above conclusions derived from plots in Figures 2B–E as a positive quality control of the obtained gene expression profiles, which supports the reliability of our experimental protocol. Since Li-Wong expression summaries preserve the correlation between samples better than RMA summaries on our data set, all further analysis was performed on Li-Wong summaries.

### Detection of differentially expressed genes

Three different algorithms for detecting DE genes were used: limma [18], Cyber-T [19] and SAM [20]. Each method produces a regularized t-statistic from which a false discover rate (FDR) can be calculated to identify the most likely DE genes.

**Figure 3:**
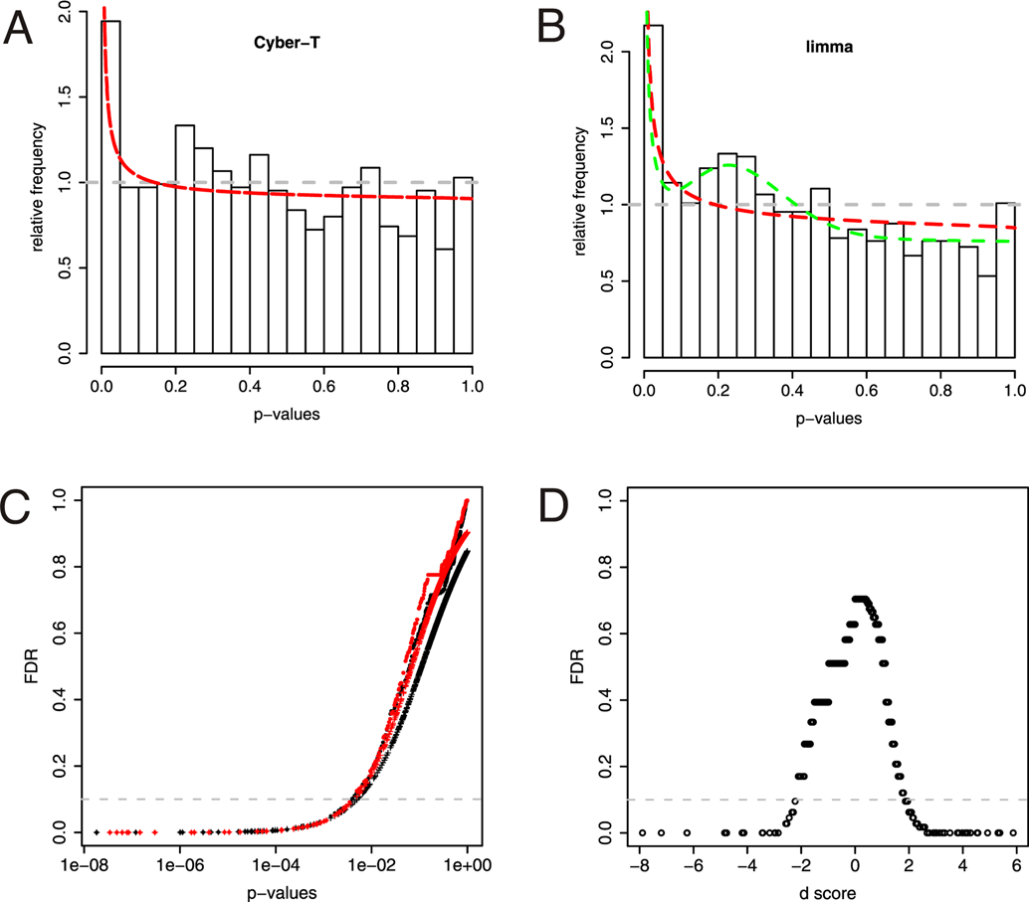
False Discover Rates. A and B display the histogram of p-values based on the regularized test statistics from limma or Cyber-T respectively when comparing MNs with dCINs. Horizontal dashed line corresponds to the uniform distribution of p-values under the null hypothesis of no differential expression. A. Mixed model fit to Cyber-T p-values, one beta distribution (red): (λ_0_, λ_1_, r, s) = (0.904, 0.0960, 0.206, 1.570). Regressions that included two or three beta-functions gave λ_2_ = λ_3_ = 0. B. Mixed model fit to limma p-values, one beta distribution (red): (λ_0_, λ_1_, r, s) = (0.848, 0.152, 0.296, 1.636) and two beta distributions (green): (λ_0_, λ_1_, λ_2_, r_1_, s_1_, r_2_, s_2_) = (0.760, 0.117, 0.122, 0.265, 2.245, 3.900, 10.042). C. Log transformed p-values versus the FDR for limma (black) and Cyber-T (red) for two different measures of FDR. Plot symbols: +represents mixture model FDR based on the fit to one beta distribution parameters from A or B and closed circles are BH FDR. D. Empirical FDR from SAM as a function of its t-statistic (d-score). Horizontal dashed line in C and D correspond to a 10% FDR cutoff.

Based on a mixed model fit to the p-values a FDR can be calculated for both limma and Cyber-T. Figures 3A and B show the histogram of Cyber-T and limma p-values respectively. Both histograms show a peak at low p-values as expected for a distribution of few DE genes on a background of non-DE genes. The horizontal grey dashed line indicates the distribution of p-values under the null hypothesis of no DE genes. Mixed model fit to Cyber-T p-values with one, two or three beta functions showed no significant difference, so only the one beta distribution fit is included (red dashed curve in Figure 3A). The mixed model fit with two beta distribution for the limma p-values does on the other hand introduce an additional peak (green dashed curve in Figure 3B). This indicates that the limma p-values have some dependence structure that is not present to the same extend in the Cyber-T p-values.

The FDRs were estimated from the mixed model regression parameters with one beta distribution (red dashed curves in Figures 3A and B) and can be seen as a function of the log-transformed p-values in Figure 3C for limma (black) and Cyber-T (red). These curves illustrate the mapping between p-values and FDRs, e.g. for a 5% FDR the corresponding p-value cutoff becomes approximately 0.2%. For comparison the FDR based on Benjamini and Hochberg (BH) [23] is also plotted in Figure 3C. Both FDRs produce similar curves that primarily deviate at high FDR. There seems to be little difference whether BH or the mixed model approach is used to estimate FDR for limma and Cyber-T on our data set, but since the BH FDR produce values that tend to increase stepwise whereas the mixed model have a more smooth increase (more obvious if Figure 3C is plotted in higher resolution, not shown) we chose to use the mixed model FDR in the following. For SAM the empirical FDR is plotted as a function of the t-statistic (the d-score), Figure 3D. A significance cutoff level of 10% for the FDR is illustrated as a horizontal dotted line in Figures 3C and D. The number of DE genes at this level of significance corresponds to the amount of points falling below this line: Cyber-T 40, limma 44 and SAM 76 DE genes. This gives an average of 53 DE genes at the 10% FDR level. Cyber-T and limma produce similar number of DE genes, whereas SAM classifies almost twice as many genes as DE at each level of significance. Thus SAMs empirical FDRs seem less conservative than FDRs based on either Cyber-T or limma. We therefore set our threshold for DE at 50 genes, slightly biasing the average towards that of Cyber-T and limma.

To obtain a conglomerate estimate of the likelihood of DE, all genes were ranked according to the absolute value of their regularized t-statistic (ranking according to p-values or FDRs would give the same order of DE genes) for each of the three methods separately and a list of genes was produced based on the average rank of all three methods. The 50 most DE genes from this list are shown in Table 1. In general there is very good agreement between all three methods as the rankings for each gene fall quite close for all three DE classification methods. From Table 1 it is also clear that SAMs empirical FDR is quite coarse grained as it increases in steps rather than continuously, which is also evident from Figure 3D. The FDR is displayed in Table 1 for each of the three methods and the majority of the DE genes have at least one of the three FDRs falling below 5%.

**Table 1:** Ranking of the 50 most differentially expressed genes.

Affy ID	Gene Symbol	Rank Cyber-T	Rank Limma	Rank SAM	Rank Avg	FDR Cyber-T	FDR Limma	FDR AM	A	M
AF041246_at	OX2R	1	5	5	3.7	5.04E-05	0.0605	0	5.79	7.2
S49491_s_at	PENK	3	7	7	5.7	0.00129	0.101	0	6.17	3.32
U89608_at	EAA4	9	6	6	7	0.0221	0.0787	0	7.15	2.79
AF030253_at	VIAAT	6	9	8	7.7	0.00202	0.249	0	7.77	5.29
M83561_s_at	GRIK1	8	10	12	10	0.0055	0.271	0	5.45	2.14
AF041244_at	OX1R	12	11	11	11.3	0.0784	0.304	0	6.93	2.53
L10073_at	5HT5B	13	16	19	16	0.0934	0.47	0	5.51	1.66
X04139_s_at	KPCB	20	17	15	17.3	0.696	0.624	0	9.18	2.61
AF058795_at	GABR2	17	19	17	17.7	0.384	0.682	0	7.77	2.72
M61099_at	MGR1	10	23	22	18.3	0.0248	1.63	0	5.78	4.28
AF030358_g_at	X3CL1	27	20	20	22.3	2.26	0.735	0	10.8	2.04
M90518_at	Q62916	29	21	21	23.7	2.57	1.02	0	8.42	2.23
M93273_at	SSR2	25	29	29	27.7	1.8	3	0	5.94	1.61
rc_AI228669_at	SC6A1	18	36	31	28.3	0.391	6.6	0	6.68	3.82
M15880_at	NPY	36	26	26	29.3	6.08	1.79	0	5.92	1.41
L05435_at	SV2A	23	35	32	30	1.52	6.2	0	7.91	2.94
X55812complete_seq_at	CNR1	19	38	35	30.7	0.678	6.74	0	6.75	3.06
rc_AI228113_s_at	NPTXR	22	34	36	30.7	1.29	6.08	0	6.15	2.11
X04979_at	APOE	49	22	23	31.3	14.2	1.37	0	12.2	1.61
U08290_at	NNAT	71	12	13	32	28.1	0.314	0	9.56	1.51
X62840mRNA_s_at	KCNC1	43	30	30	34.3	11.8	4.35	0	7.57	1.84
M38061_at	GRIA2	26	41	40	35.7	1.86	8.64	0	5.96	1.87
M32867_at	KCNA4	24	45	47	38.7	1.52	10.4	1.74	4.94	1.64
S82649_s_at	NPTX2	48	32	37	39	13.9	5.62	0	7.99	1.88
L14851_at	NRX3A	35	47	41	41	5.52	11.8	1.74	7.89	2.48
M31174_at	THA	69	27	27	41	26.5	2.23	0	11.08	1.51
rc_AI177026_at	AT1A2	31	53	44	42.7	2.99	13.2	1.74	7.71	3.06
rc_AA957510_s_at	AT2A2	56	39	39	44.7	18	6.95	0	9.11	1.74
U16845_at	NTRI	28	58	49	45	2.53	16.3	1.74	6.22	2.97
D00833_g_at	GLRA1	46	48	54	49.3	12.2	12	2.8	4.41	1.32
M24852_at	PEP19	34	64	53	50.3	4.98	19.4	2.8	9.35	3.75
S76779_s_at	APOE	65	43	43	50.3	25.3	9.8	1.74	8.06	1.72
E12625cds_at	ERG25	51	49	55	51.7	14.9	12	2.8	4.96	1.25
rc_AI029920_s_at	IBP5	38	55	52	48.3	7.15	13.8	6.21	5.78	−1.59
U03491_at	TGFB3	42	44	45	43.7	11.8	9.91	2.8	6.34	−1.61
M84725_at	TAGL3	53	33	38	41.3	16.9	5.63	0	9.9	−1.71
rc_AI008865_s_at	STAT3	47	31	34	37.3	12.8	4.96	0	8.52	−1.85
AF016296_at	NRP1	16	37	33	28.7	0.33	6.61	0	5.16	−3.55
U01227_s_at	5HT3R	37	24	24	28.3	6.88	1.68	0	6.48	−1.68
rc_AA998683_g_at	HSPB1	30	25	25	26.7	2.64	1.72	0	6.09	−1.65
M27925_at	SYN2	21	28	28	25.7	1.09	2.51	0	5.15	−1.48
M64488_at	SYT2	33	15	18	22	3.68	0.415	0	6.17	−1.56
X12589cds_s_at	KCNA1	32	14	16	20.7	3.38	0.39	0	6.03	−1.52
M60654_at	ADA1D	11	18	14	14.3	0.0435	0.637	0	6.01	−2.75
rc_AA818677_at	NFH	15	8	10	11	0.272	0.199	0	11.02	−2.43
U09211_at	VACHT	4	13	9	8.7	0.00158	0.346	0	10.21	−11.07
AF031880_at	NFL	14	1	1	5.3	0.142	0.00165	0	11.31	−2.3
X05137_at	TNR16	5	3	4	4	0.0019	0.0278	0	8.16	−4.51
M11596_at	CALCB	7	2	2	3.7	0.00311	0.00622	0	7.59	−3.93
X86789_at	SYUG	2	4	3	3	0.000989	0.0302	0	8.85	−5.34

A = average log_2_ transformed expression; M = log_2_ transformed average ratio.

Several genes on the RN_U34 chip is represented by two or more probe sets. This is the case for the gene *Apoe* which is represented by two probe sets that both appear in the list of the 50 most DE genes in Table 1. So in fact the unique set of genes in Table 1 only add up to 49 and not 50.

### Validation of differentially expressed genes

Among the unique DE genes displayed in Table 1, a representative subset of seven genes were chosen for micorarray validation with real time reverse transcription PCR (real time RT-PCR); MN>dCIN: *Nefl, Calcb, Kcna1, 5HT3r, VAChT*; dCIN>MN: *Ox2r, Viaat*. *VAChT* (vesicular acetylcholine transporter) and *Viaat* (vesicular inhibitory amino acid transporter, GABA and glycine release) were expected a priori to be DE between MNs and dCINs [15] and were chosen for this reason. *Ox2r* (orexin receptor 2), *Calcb* (calcitonin gene-related peptide) and *Nefl* (neurofilament light chain) were not expected a priori to be DE and were chosen for validation to make sure they were not false positives. There is presently no consensus regarding whether differences in ion channels and receptors, that constitute the major functional differences between neurons, are subject to active regulation at the transcriptional level. We therefore chose the gene for one receptor, the serotonin receptor 5-HT_3A_ (*5HT3r*), and the gene for one ion channel, the potassium voltage-gated channel subfamily A member 1 (*Kcna1*, synonymous with *Kv1.1*), for validation to establish whether these were indeed true positives.

**Figure 4:**
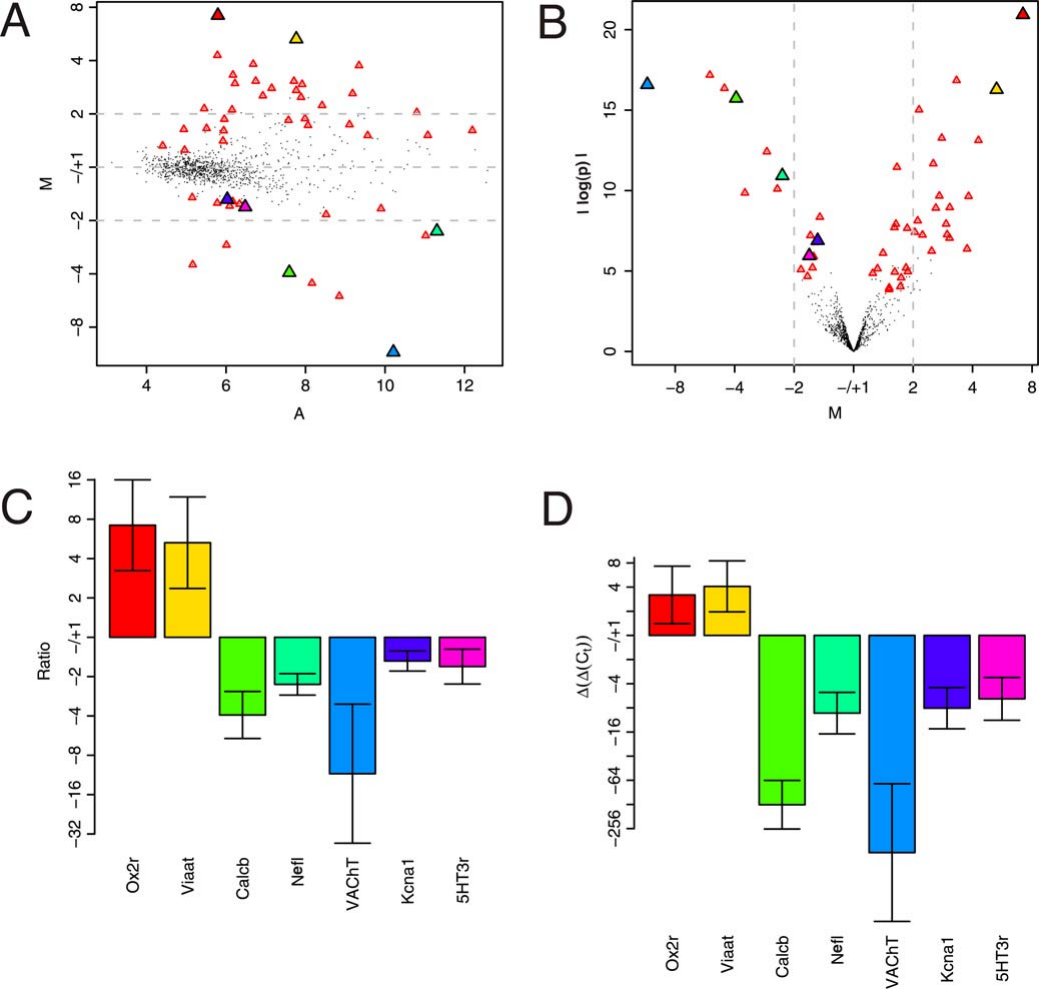
Differentially expressed genes. Positive ratios (M in A–B and y-axis in C–D) represent higher mRNA levels in dCINs than in MNs and negative ratios represent lower mRNA levels in dCINs than in MNs. The scale of ratios is displayed in normal values such that the value of the ratios can be read directly from the figures. Red triangles in A and B highlight the 50 most significantly DE genes from Table 1. The enlarged filled triangles in A and B are the genes chosen for real time RT-PCR validation, with the same color code throughout including bar-plots C–D. A. MA plot of MNs versus dCINs, i.e. for each gene the log_2_ transformed average ratio, M (*eq 5* and *eq 6*), is plotted as a function of the average log_2_ transformed expression values, A (*eq 7*). B. Volcano plot of M (same as in A) versus the absolute log transformed p-values from Cyber-T. C. Bar-plot of log_2_ transformed ratios (M values from A and B) of the genes chosen for real time RT-PCR. The genes are plotted in decreasing order of significance of DE within each group, dCINs and MNs. The error bars indicate the standard deviation of the log_2_ transformed ratios. D. Barplot of log_2_ transformed ratios (ΔΔC_T_) from the real time RT-PCR validation of the same genes as in C. Color codes for the highlighted real time RT-PCR genes match on A–D: red = *Ox2r*, yellow = *Viaat*, dark green = *Calcb*, light green = *Nefl*, blue = *VAChT*, purple = *Kcna1*, pink = *5HT3r*.

The ratios of expression of the real time RT-PCR validated genes are illustrated both for the microarray data and the real time RT-PCR counterpart in Figure 4. First, in order to illustrate how the ratios of expression of the DE genes are distributed among the total set of genes, the 49 most DE genes from Table 1 are highlighted as red triangles in two standard plots: the MA (Figure 4A) and the volcano (Figure 4B) plot. The 7 validation genes contained within the 49 most DE genes are further highlighted as enlarged triangles in different colors. In the MA plot we summarize the differences in gene expression between the two experimental groups, MNs and dCINs, by plotting the log_2_ transformed values of the average ratio between the two groups, M (*eq 5* and *eq 6* in Materials and Methods), as a function of the average log_2_ transformed expression values, A (*eq 7* in Materials and Methods). In the volcano plot M is plotted as a function of significance, in this case the absolute log transformed p-values from the Cyber-T test. A plot using limma p-values or SAM FDRs shows similar characteristics (not shown). The grey dotted lines indicate a twofold ratio of expression in both the MA and the volcano plot. Positive ratios represent more expression of respective genes in dCINs compared to MNs and negative ratios more expression of respective genes in MNs compared to dCINs. From Figures 4A and B it can be seen that the 7 genes chosen for validation fall throughout the range of expression values A and ratios M among the 49 most DE genes and thus constitute a good representation of the genes in Table 1. It can be noted from both the MA and the volcano plot that significantly DE genes can have quite low expression ratios as some of the most significantly DE genes exhibit less than a twofold difference, an otherwise commonly used ratio cutoff level of significance. *Kcna1* and *5HT3r* both fall into this category.

From Figure 4A the average ratio for the seven validation genes were extracted and replotted in Figure 4C, which also illustrates the standard deviation of each gene (error bars). For the same genes Figure 4D shows the real time RT-PCR ratios of expression between MNs and dCINs with respect to the housekeeping gene *Gap-dh*, displayed as Figure 4C. The color codes for the validated genes are the same in all four plots (Figures 4A–D). All seven genes show good agreement between microarray and real time RT-PCR ratios. A similar pattern of differential gene expression was seen for real time RT-PCR ratios with respect to the housekeeping gene *Beta-actin* (not shown).

### Biological function of differentially expressed genes

To assess the biological relevance of the DE classified genes, the DE genes from Table 1 were reorganized into functional classes and displayed accordingly in Table 2. We divided the genes into seven functional groups: 1) voltage-gated channels and ion pumps, 2) receptors and ligand gated channels, 3) transporters and transmitter release, 4) growth factors and axon guidance, 5) axonal and cytoskeletal structure, 6) cell adhesion and 7) transcriptional regulation. Genes not fitting into any of these categories were lumped together into a separate group termed miscellaneous. Seventeen genes were more expressed in MNs compared to dCINs and 32 genes were more expressed in dCINs compared to MNs.

**Table 2:** Functional classification of the 50 most differentially expressed genes.

	MNs>dCINs			dCINs>MNs		
	Gene ID	Affy ID	Description	Gene ID	Affy ID	Description
**Voltage-gated channels & ion pumps**	*Kcna1*	X12589cds_s_at	Potassium channel (Kv1.1, Shaker-related subfamily 1)	*Kcnc1*	X62840mRNA_s_at	Potassium channel (Kv3.1, Shaw-related subfamily 1)
				*Kcna4*	M32867_at	Potassium channel (Kv1.4, Shaker-related subfamily 4)
				*At1a2*	rc_AI177026_at	ATPase of Na+/K+ pump
**Receptors & ligand gated channels**	*Ada1d*	M60654_at	Adrenergic alpha-1D receptor	*Grik1*	M83561_s_at	Kainate receptor (GluR5)
	*5HT3r*	U01227_s_at	5-HT_3A_ receptor	*Gria2*	M38061_at	AMPA receptor 2 (GluR2)
				*Q62916*	M90518_at	Metabotropic glutamate receptor 4b (mGluR4)
				*Mgr1*	M61099_at	Metabotropic glutamate receptor 1 (mGluR1)
				*Glra1*	D00833_g_at	Glycine receptor alpha-1 subunit
				*5HT5b*	L10073_at	5-HT_5B_ receptor
				*Ox1r*	AF041244_at	Hypocretin (orexin) receptor type 1
				*Ox2r*	AF041246_at	Hypocretin (orexin) receptor type 2
				*Ssr2*	M93273_at	Somatostatin receptor type 2
				*Cnr1*	X55812complete_seq_at	Cannabinoid receptor 1
				*Gabr2*	AF058795_at	GABA_2B_-receptor
**Transporters & transmitter release**	*VAChT*	U09211_at	Vesicular acetylcholine transporter	*Viaat*	AF030253_at	Vesicular inhibitory amino acid transporter
	*Syn2*	M27925_at	Synapsin II	*EAA4*	U89608_at	Aspartate/glutamate transporter 4
	*Syt2*	M64488_at	Synaptotagmin-2	*At2a2*	rc_AA957510_s_at	Endoplasmic reticulum calcium ATPase 2
				*SC6A1*	rc_AI228669_at	Sodium- and chloride-dependent GABA-
						transporter 1 (Gat1)
				*Sv2a*	L05435_at	Synaptic vesicle protein 2
				*Nrx3A*	L14851_at	Neurexin-3-alpha
**Growth factors & axon guidance**	*Ibp5*	rc_AI029920_s_at	Insulin-like growth factor binding protein 5			
	*Tnr16*	X05137_at	Neurotrophin receptor (p75NTR)			
	*Tgfb3*	U03491_at	Transforming growth factor beta-3 precursor			
	*Nrp1*	AF016296_at	Neuropilin-1 (semaphorin co-receptors)			
**Axonal & cytoskeletal structure**	*Nefh*	rc_AA818677_at	Neurofilament heavy chain (NFH)			
	*Nefl*	AF031880_at	Neurofilament light chain (NFL)			
	*Syug*	X86789_at	Gamma synuclein			
	*Hspb1*	rc_AA998683_g_at	Heat shock 27kDa protein beta-1			
	*Tagl3*	M84725_at	Transgelin 3, Neuronal protein NP25 or NP22 (actin)			
**Cell adhesion**				*NtrI*	U16845_at	Neurotrimin (*Hnt*)
**Transcriptional regulation**	*Stat3*	rc_AI008865_s_at	Signal Transducers and Activators of Transcription 3	*Thra*	M31174_at	Nuclear thyroid hormone alpha receptor (c-erbA-1)
			(Cytokine-responsive genes)			
**Miscellaneous**	*Calcb*	M11596_at	Calcitonin gene-related peptide	*Nnat*	U08290_at	Neuronatin (transmembrane protein)
				*Pep19*	M24852_at	Calmodulin-binding peptide 19
				*ERG25*	E12625cds_at	Neuropep-1 (methyl sterol oxidase)
				*Apoe*	X04979_at	Apolipoprotein E
				*Kpcb*	X04139_s_at	Protein kinase C beta type
				*Nptxr*	rc_AI228113_s_at	Neuronal pentraxin receptor
				*Nptx2*	S82649_s_at	Neuronal pentraxin 2 (*Narp*)
				*Npy*	M15880_at	Neuropeptide Y
				*X3cl1*	AF030358_g_at	Fractalkine precursor (chemokine)
				*Penk*	S49491_s_at	Pro-enkephalin

Of the 17 genes DE in MNs all but 1 have previously been described in this cell population in rodents. The exception is a gene involved in cytoskeletal structures, *Tagl3*, otherwise only described in brain neurons [24], [25]. Among the DE genes that correlate with known MN function we note that MNs: release acetylcholine (*VAChT*); receives adrenergic (*Ada1d*) and serotonergic (*5HT3r*) input [26], [27]; have an extensive cytoskeleton, which is reflected by the fact that 5 of the 17 DE genes are in this category i.e. the genes for neurofilament heavy chain (*Nefh*) [28], neurofilament light chain (*Nefl*) [28], gamma synuclin (*Syug*) [29], heat shock protein beta-1 (*Hspb1*) [30], [31] and transgelin-3 (*Tagl3*). The genes for transforming growth factor beta-3 (*Tgfb3*) and neuropilin-1 (*Nrp1*) were also DE in MNs and both have been described to participate in MN development and survival [32], [33]. Additional genes more expressed in MNs that have previously been reported to be present in this population include the genes for synapsin II (*Syn2*) [34] and synaptotagmin-2 (*Syt2*) [35] both involved in synaptic release and its regulation, a potassium voltage-gated channel (*Kcna1*, synonymous with *Kv1.1*) [36]–[38], a calcitonin gene-related peptide (*Calcb*) [39], the insulin-like growth factor binding protein 5 (*Ibp5*) [40], a neurotrophin receptor (*Tnr16*) [41] and a transcription factor (*Stat3*) [42].

There is presently little work on gene expression of dCINs [43], but of the 32 genes classified as DE in the dCINs in our study all but 2 genes have previously been reported to be present in the spinal cord and among these only 1 gene, *Viaat*, has been shown to be specifically linked to dCINs [44]. The two genes DE in dCINs not previously described in the spinal cord constitute a gene coding for a nuclear thyroid hormone alpha receptor (*Thra*) and a gene coding for a serotonin receptor (*5HT5b*). Serotonin receptors are present in the spinal cord CINs [45], but the 5-HT_5B_ receptor (*5HT5b*) has not been shown to be expressed in the spinal cord. *Thra* has been linked to development and maturation of GABAergic cells in the neocortex of mice [46] and could therefore also be part of the development and maturation of the dCINs, a subpopulation of which are know to contain GABA [43]. The inhibitory nature of some of the dCINs are also reflected in the fact that two out of the three DE genes in dCINs that relate to transmitter release and reuptake codes for proteins involved in GABA and glycine release: a vesicular transporter for GABA and glycine (*Viaat*) [47] and a Na^+^/Cl^−^ dependent GABA transporter (*SC6A1*, synonymous with *Gat1*) [48]. The third gene in this category reflects that some dCINs release glutamate [15], [49] as they express the gene for a membrane transporter of aspartate and glutamate (*EAA4*) [50]. Among the known spinal cord positive genes DE in dCINs there are 10 genes coding for receptors and ligand gated ion channels: four glutamate receptors, *Grik1* (synonymous with *GluR5*) [51], *Gria2* (synonymous with *GluR2*) [51], *Q62916* (synonymous with *mGluR4*) [52] and *Mgr1* (synonymous with *mGluR1*) [52]; a glycine receptor alpha subunit (*Glra1*) [53]; a GABA receptor (*Gabr2*) [53]; two orexin receptors (*Ox1r* and *Ox2r*) [54]; a somatostatin receptor (*Ssr2*) [55] and a cannabinoid receptor (*Cnr1*) [56]. Additional genes DE in the dCIN population relating to neuronal signal transduction include genes for two voltage-gated potassium channels {(*Kcnc1*, synonymous with *Kv3.1*) [57] and (*Kcna4*, synonymous with *Kv1.4*) [58]}, an ATPase Na^+^/K^+^ pump (*At1a2*) [59] plus four genes linked to synaptic transmission: a gene involved in vesicle release, the synaptic vesicle protein 2 (*Sv2a*) [60]; a gene involved in Ca^2+^ channel and release site organization, neurexin-3-alpha (*Nrx3a*) [61]; two genes linked to glutamate receptor clustering, the peptide neuronal pentraxin 2 (*Nptx2*, synonymous with *Narp*) and its receptor neural pentraxin receptor (*Nptxr*) [62]. Two genes surprisingly found to be DE in the dCIN population codes for the opioid neuropeptide precursor pro-enkephalin (*Penk*) [63] and neuropeptide Y (*Npy*) that mainly have been implicated in pain perception [64], [65]. The ostensible function of either in the dCINs is not immediately clear (see Discussion). The last eight DE genes in the dCINs code for a mixed group of proteins: neuronatin (*Nnat*) [66] and neurotrimin (*NtrI*, synonymous with *Hnt*) [67] are involved in maturation and general maintenance of overall structures in the central nervous system; calmodulin-binding peptide 19 (*Pep19*) [68] and endoplasmatic reticulum calcium ATPase 2 (*At2a2*) [69] are involved in calcium regulation; protein kinase C beta (*Kpcb*) [70] is involved in Ca^2+^ dependent second messenger signaling; apolipoprotein E (*Apoe*) [71] and possibly methyl sterol oxidase (*ERG25*) [72], [73] are involved in lipid metabolism; the chemokine fractalkine (*X3cl1*) is involved in neuron-to-glia signaling [74].

All the 49 DE genes of Table 1 are neuron related, further indicating that potential contamination from surrounding cells such as glia during LMD is minimal. There are probe sets on the RN_U34 chip for genes that have traditionally been used as glial cell markers: glial fibrillary acidic protein (*GFAP;* probe sets AF028784cds#1_s_at and AF028784mRNA#1_s_at) and myelin basic protein (*MBP*; probe set K00512_at). Neither show any change in expression between the two groups and have very low intensity levels, *GFAP* (AF028784cds#1_s_at/AF028784mRNA#1_s_at): fold change = −1.01/−1.14, FDR = 87/74%, average intensity = 29/50 and *MBP*: fold change = 1.03, FDR = 87%, average intensity = 36. Since the dynamic intensity range of our data set is {1;8642} we conclude that there are no expression of these genes in our samples which suggest a minimal glial contamination from the LMD.

## Discussion

With the advance of the microarray platforms gene expression profiling has been widely used, especially for the characterization of homogenates from whole anatomical structures or tissue biopsies [75]–[77]. Recent technical advances have enabled the extraction of identified cell populations, which combined with the microarray platform have been used as a classification tool for neuronal cell type taxonomy [5], [6]. Along similar lines we have in the present study shown that it is possible to obtain specific gene expression profiles from fluorescently identified cell populations in the mammalian spinal cord based on microarrays hybridized with amplified aRNA from as few as 50 cells. The use of identified cell populations opens up for studies of global gene expression dynamics within distinct cell populations [78].

Using the optimized experimental protocol complemented with the microarray analysis methodology, as presented in this study, we identify 49 genes as DE between the MNs and dCINs. In the MN population 17 genes were more expressed than in the dCIN population, while 32 genes were found to be more expressed in the dCIN population compared to the MN population. The gene expression profiles of each of the two neuronal populations reflect their different anatomical and functional characteristics.

### Methodological considerations

We selected a path of analysis that, within the tested methods, led to a robust classification of DE genes on our data set. We first introduced a simple algorithm to examine and possible validate probe level distribution similarity, termed QLT. For each probe, the microarray signal consists of a combination of true gene expression signal and several sources of experimental noise, while it at the same time is subject to biological variation. Since the correct distributions of the gene expression profiles are in fact unknown, assumptions have to be made about the corresponding microarray signal structure in order to extract the true gene expression signal. First, normalization is carried out to eliminate experimental effects that cause between-chip variations, while any remaining common sources of error masking the true signal are handled in the downstream analysis following normalization. Two existing normalization methods, QPN and qspline [22], [79], minimize between-chip variation by nonlinear transformations that map all microarray probe level distributions onto the average distribution of the constituting data set. Both methods rest on the biological assumption that all transcript level distributions are conserved in spite of any experimental perturbations. Hence differences among the observed microarray probe level distributions are attributed to experimental effects and the normalization is designed to equal all these distributions. With the introduction of the QLT we make an initial normalization step that in effect separates the experimental variations into linear and nonlinear parts. The QLT transformation accomplish this by rescaling each array using parameters from a linear regression between quantiles of the probe level distributions and the average probe level distribution, leaving nonlinear variations unaffected. Visual inspection of the QLT distributions can be used to illustrate the degree of linear and nonlinear experimental variations in the data set given the assumption of equal gene expression distributions. This means that pure linear experimental variations produce identical overlapping QLT distributions whereas additional nonlinear variations will show up as deviations of the QLT distributions from the average distribution. This transformation could therefore potentially suffice to produce identical intensity distributions if there are no nonlinear experimental variations. In the case of predominant linear experimental variations we also find it more plausible to assume that the actual gene expression profiles are in fact identical. The QLT can furthermore be used as a visualization tool to inspect array quality and effects of background compensation. Array outliers will typically show up as deviating distributions among the set of identical distributions. We found no array outliers, in fact all 22 QLT distributions superimposed validating the basic assumption that the microarray distributions are identical in our data set (Figure 2B). Having confirmed the convergence of the experimental microarray distributions we further tested the effect of additional QPN following QLT to compensate for minor nonlinear effects at the distribution tails. We found that QLT followed by QPN [80] improved the correct clustering of samples according to experimental groups (MNs, dCINs and MIX) compared with either normalization procedure used alone. On our data set this effect of normalization seemed to have the biggest influence on RMA expression profiles, indicating that the pattern of expression is better preserved in Li-Wong expression summaries than in RMA summaries. We therefore based the DE classification methods on the Li-Wong expression summaries.

Three methods were used to classify genes as DE within a 10% FDR: Cyber-T, limma and SAM. All three methods agree quite well with each other, but in order to increase the consistency of DE detection a conglomerate classifier based on the average rank of all three statistical tests was used. With a FDR cutoff of 10% for each method we find that an average of 49 genes are significantly DE. In other words less than 5% of the neuron related genes on the microarray. It is worth noting that although we chose an initial cutoff of 10% for each method, using the conglomerate classifier presented here has the effect that the majority of genes in fact have at least one FDR value that fall below 5%. The combination of three methods to detect DE genes thus gives candidates that have lower FDRs than would be expected from the actual cutoff of each individual method. The variation in gene ranks (and FDRs) between the three methods can possibly be attributed to the differences in how they attempt to overcome instabilities in the gene specific variance estimates. Especially any variance-to-mean dependence remaining after the log_2_ variance stabilizing transformation will affect the regularized t-statistic when this is based on the assumption of homoscedasticity (i.e. homogeneity of variance), which is the case for limma and SAM. Cyber-T is the only of the three methods that incorporates this sort of dependence into its estimate of the t-statistic. This could also be the reason why the p-value histogram of Cyber-T shows less dependence than limma p-values (Figures 3A and B). Interestingly this dependence structure for limma p-values seems to be reduced in the RMA expression profiles (Supplementary Figure S1).

In summary, we conclude that the combined effect of having three FDRs for each gene may help to determine DE genes. We also note that the relative high number of seven to eight replicates within each experimental group improves the power of each test to detect DE genes. From the list of the 49 most DE genes 7 representative genes were chosen for real time RT-PCR validation and all confirmed the findings of the microarrays.

### Biological significance of differential gene expression

Among the 49 most DE genes in this study all have previously been described in neurons. Most of these genes have been reported in either MNs or the spinal cord in general, supporting the quality of our microarray data. All the genes DE in MNs have previously been found in this cell class except *Tagl3*, a gene involved in cytoskeletal structures [24], [25]. Except for *Viaat* [44], the 32 genes found to be DE in the dCINs have never been associated with this particular neuronal population. Together our findings suggest that functional differences between MNs and dCINs are reflected at the level of gene expression.

At the anatomical level MNs are larger in diameter and they generally have longer axonal projections than dCINs, therefore it is perhaps not surprising that there is an over-representation of cytoskeletal related DE genes in the MN population. Five of the 17 DE genes belonging to the MNs fall into this category whereas none do so for the DE genes in the dCINs. The two cell populations are also known to release different neurotransmitters, which is confirmed by the microarray results. MNs release acetylcholine supported by the DE of *VAChT* in this population. The dCINs on the other hand constitute a mixed population of both inhibitory and excitatory cells that release either GABA/glycine or glutamate [15], [43], [49], reflected by the DE of *Viaat*, *Gat1* and *EAA4* in these cells. Two genes, *Npy* and *Penk*, coding for neuropeptides that previously have been connected mainly with the regulation of pain perception in the spinal cord, were surprisingly found to be DE in the dCIN population. Pro-enkephalin immunoreactive fibers have been described in motor columns and close to the central canal [63]. However, there are no previous studies showing mRNA expression of *Penk* in interneurons in the ventral spinal cord, although in this study the expression of *Penk* was ranked high with all algorithms used for detection. Neuropeptide Y (*Npy*) on the other hand has been described in interneurons located in the intermediate part of the spinal cord [81], but it has also been found to be transiently expressed there and in lamnia X prenataly [82].

Among the observed differences that may relate to functional specialization in neuronal firing and/or synaptic integration are the diverse expression of genes coding for three voltage gated potassium channels in MNs and dCINs: *Kv1.1* is more expressed in MNs whereas *Kv3.1* and *Kv1.4* are more expressed in dCINs. *Kv1.1* and *Kv3.1* both codes for delayed rectifying channels, where the channel encoded by *Kv3.1* is activated at more depolarized membrane potentials and inactivates faster than the channel encoded by *Kv1.1* [83]. *Kv1.4* codes for an A-type fast-inactivating potassium channel that has been shown to induce rapid repolarization of the action potential [83]. These properties of the channels encoded by *Kv3.1* and *Kv1.4* suggests that dCINs can produce faster spiking [84], [85] than MNs although such functional tests have not been done systematically in the rodent spinal cord.

It furthermore seems that the dCINs have a more dense and broader range of receptors/ligand gated channels than the MNs, which will affect the synaptic integration in the two cell populations. There are 11 genes in this category that are more expressed in dCINs than in MNs, while there are only 3 genes in this category that are more expressed in MNs than in dCINs. The analysis shows that the two cell populations differ in the gene expression of two different types of 5-HT receptors and an alpha-1 adrenoreceptor (*Ada1d*). The gene for the ionotropic 5-HT_3A_ receptor (*5HT3r*) is more expressed in the MNs as is the gene for the alpha-1D adrenoreceptor (*Ada1d*), while dCINs express more of the gene coding for the metabotropic 5-HT_5B_ receptor (*5HT5b*). Expression of *5HT5b* has not previously been described in the spinal cord. The consequences of this differential distribution may reflect functional differences in the response to descending serotonergic and noradrenergic signals from the brainstem [45]. The additional DE genes for receptors in the dCIN population reflect the fact that dCINs integrate various synaptic signals to coordinate left-right movement during locomotion. Ionotropic receptor channels for glutamate and glycine (*GluR5*, *GluR2* and *Glra1*) are strongly activated during rhythmic activity such as locomotion [14], whereas some of the metabotropic receptors are know to cause slow modulation of the activity (*mGluR1* [86], *Gabr2* [87] and *Cnr1* [88]). Obviously some of these receptors, like the receptors for AMPA, kainate, glycine and mGluR1, are also found in MNs [89]. However, our study suggests that the MNs achieve a synaptic integrative function by a less dense distribution of these receptors than found in dCINs.

The genes coding for orexin and somatostatin receptors were DE in dCINs. Orexin has been suggested to be involved in sensory modulation, as it has been found to have a high expression in both pre-ganglionic sympathic cell columns and descending fibers terminating around the central canal close to the location of the dCINs [90]. The DE of the genes coding for both orexin receptor 1 and 2 in the dCINs indicate that these cells could be a target for such sensory modulation and integration. Somatostatin is another molecule connected with sensory input and its modulation, in particular nociception [91]. Somatostatin is expressed in laminae I-III of dorsal horn cells [92], [93], whereas its receptor has been found to be expressed in several parts of the spinal cord including the ventral cord where the dCINs are located [93]. The apparent DE of the gene coding for somatostatin receptor 2 (*Ssr2*) in dCINs could imply a role for this population in sensory integration and modulation.

### Conclusions

The protocol developed in this study was used to describe the static differences in transcript levels between two identified populations of neurons in the lumbar spinal cord of the neonatal rat, the MNs and dCINs. Our findings constitute a step forward towards understanding the functional differences between the MNs and the dCINs in the spinal cord. We also describe a new probe-level pre-processing analysis that illustrates the linear relationship between probe intensity distributions. These linearly transformed distributions can be used as an initial microarray quality assessment. This analysis method also helps the user to determine the distributional effect of background compensation and normalization and can thus help to guide the choice of these.

The experimental protocol described here furthermore has a much wider range of application. Long-term changes in electrical properties due to external perturbations, in particular spinal cord injury, have been reported in MNs [94]–[96]. Such activity dependent changes in membrane and cellular properties are arguably reflecting underlying modulation of gene expression, but to pinpoint the molecular mechanisms and targets of these changes have until recently remained elusive. In traditional microarray gene expression studies of spinal cord injury, tissue homogenates of whole spinal cord segments have been used [75], [76], [97], [98] and they therefore do not address the dynamics of global gene expression regulation in individual cell populations [75], [76], [97]. The present study provides a robust experimental protocol and an analysis methodology that enables such examinations at the cellular level and can thus be seen as an important step to examine changes in cell specific gene expression profiles over time, as for example in identified neurons after spinal cord injury or during development.

## Materials and Methods

### Spinal cord dissection, neuron labeling and cryo sectioning

Neonatal (postnatal day 0–4) Wistar rats were used in this study. All animals were cared for and used in accordance with the directives of the local ethical committee on animal experiments, the Swedish Animal Welfare Agency and EU. The rats were anesthetized with isofluran (Forene®; Abbott Scandinavia), decapitated, eviscerated and then transferred to a dissection chamber filled with oxygenated (5% CO_2_ in O_2_) and ice-cold low calcium Ringer's solution [111,14 mM NaCl/3,09 mM KCl/11,10 mM Glucose/25 mM NaHCO_3_/3,73 mM MgSO_4_/1,10 mM KH_2_PO_4_/0,25 mM CaCl_2_ (all from Merck) in 0,1% (v/v) Diethylpyrocarbonate-treated (DEPC; Sigma) and autoclaved dH_2_O; pH 7,4]. The spinal cords were dissected out by removing the vertebrate bodies and all dorsal and ventral roots were cut at the base, except the L1–L6 ventral roots. MNs or dCINs in the L2 segment of the isolated spinal cord were labeled with the retrograde fluorescent tracer RDA (3000 MW; Molecular Probes) applied to the cut ends of the L2 ventral roots (MNs) or the cut end of the hemi-segmental transection of the spinal cord between L3 and L4 (dCINs) as described previously [13], Figures 1A and B. After application of RDA the spinal cords were incubated in oxygenated (5% CO_2_ in O_2_) normal calcium Ringer's solution (111,14 mM NaCl/3,09 mM KCl/11,10 mM Glucose/25 mM NaHCO_3_/1,26 mM MgSO_4_/1,10 mM KH_2_PO_4_/2,52 mM CaCl_2_ in DEPC-treated and autoclaved dH_2_O; pH 7,4) at room temperature in a dark chamber for three hours. Following the incubation the spinal cords (L1–L6 segments) were snap-frozen with CO_2_ and stored at −0°C until sectioning. The L2 segments were sectioned in a cryostat into 10 µm sections, which were mounted on nuclease and human nucleic acid free 0,9 µm POL-membrane frame slides (Leica Microsystems) and stored at −80°C until LMD.

### Laser microdissection and cell extraction

The RDA positive neurons (MNs or dCINs) were isolated from the spinal cord sections using the Leica AS LMD laser microdissection system (Leica Microsystems) at room temperature. Laser microdissected cells were collected in the cap of a PCR tube by the force of gravity and incubated in 10 µl extraction buffer (PicoPure™ RNA Isolation Kit; Arcturus) at +42°C for 30 minutes. The cell extracts were stored at −80°C until the RNA was isolated. To reduce RNA degradation the LMD procedure never exceeded one hour before extraction buffer was added to the microdissected cells. 50–250 labeled neurons (on average a 100 cells) were laser microdissected per preparation. Control samples (MIX) were generated from randomly laser microdissected cells from the ventral part of the L2 spinal cord sections.

### Total RNA isolation, mRNA amplification and aRNA biotinylation

The PicoPure™ RNA Isolation Kit was used to isolate total cellular RNA from the laser microdissected cells. A DNAse treatment was always added to the RNA isolation protocol to eliminate genomic DNA contamination (RNase-Free DNase Set; Qiagen). The mRNA fraction of the total cellular RNA was amplified by a two round T7 linear amplification process using the RiboAmp® HS RNA Amplification Kit (Arcturus). The complementary DNA (cDNA) from the second round of the amplification process was used to generate biotin-labeled aRNA in an in vitro transcription (IVT) reaction using the GeneChip® Expression 3′-Amplification Reagents for IVT Labeling (Affymetrix). The yield and purity of the biotinylated aRNA samples were determined in a BioPhotometer (Eppendorf) and only samples of good integrity were further processed. RNA isolations including DNase treatment, RNA amplifications and biotin-labelings were performed according to manufacturers' instructions.

We amplified mRNA from 50–250 laser microdissected cells for microarray hybridization, keeping the input material within a range of a factor five as suggested in [5] and therefore minimizing complications in downstream analysis due to amplification artifacts [5], [99].

### RNA quality control and microarray hybridization

The integrity of the biotinylated and amplified aRNA samples were furthermore assessed on Agilent RNA chips with the Agilent 2100 Bioanalyzer (Agilent Technologies), both before and after fragmentation of the samples. Five µg of the fragmented samples were hybridized to RN_U34 chips, which were subsequently scanned. One array always originated from one animal. The Agilent quality controls and microarray hybridizations were done by the Affymetrix core facility at Novum (Bioinformatics and Expression Analysis core facility, Department of biosciences and nutrition, Karolinska Institutet, Huddinge, Sweden) according to the manufacturers' instructions.

### Real time reverse transcription PCR

Real time RT-PCR was used to validate the microarray hybridization results for seven of the genes detected as DE. The real time PCR was performed on one linear round amplified cDNA according to the microarray protocol above to facilitate direct comparison with the microarray hybridization results. As normalization genes we used the housekeeping genes *Gap-dh* and *Beta-actin*, which both showed little variation in expression across samples on the microarrays. The sequences of the primers are listed in Table 3. The real time PCR was performed using the ABI Prism® 7000 Sequence Detection System (Applied Biosystems) and the SYBR® Green PCR Master Mix (Applied Biosystems). The cDNA was first denatured at +95°C for 10 minutes and then the reaction profile was subjected to 45 cycles of amplification. Each cycle consisted of denaturation at +95°C for 15 seconds and annealing/extension at +60°C for 60 seconds. After the last amplification cycle, a dissociation curve was constructed by increasing the temperature from +60°C to +95°C. Four individual MN samples were compared with three dCIN samples. The limited amount of material from each sample constrained the amount of replicas to two per sample and primer set. The variation seen in the real time RT-PCR ratios (Figure 4D) therefore appear rather large, as it reflects biological variation between animals rather than experimental variations between multiple replicas of primer samples. In order to increase the reliability of the biological validation, the real time RT-PCR was performed on RNA samples originating from a separate set of animals distinct from the ones used for the microarray hybridization experiments.

**Table 3:** Real time RT-PCR primer sequences.

Gene	Oligonucleotides (5′to 3′)
**MNs>dCINs:**	
*Nefl*	
–Forward primer	GCA GAG TAT CTG TTT GCT TGC
–Reverse primer	GTG ATT CAC ATT GCC GTA GAT
*VAChT*	
–Forward primer	GTG TTA GGC GTC TAC CTC ACC
–Reverse primer	AAG AGC TCA CTC CAA TTA CCG
*Calcb*	
–Forward primer	GGA AAA CAC CAT TGT CAC TTG
–Reverse primer	TTT GAC TGG CCA TAG ACT CAG
*Kcna1*	
–Forward primer	ATG TAC CCT GTG ACA ATT GGA
–Reverse primer	GAA ATT GGA CAC AAT GAC AGG
*5HT3r*	
–Forward primer	CCT TTT TGA TCA GAG GAA AGC
–Reverse primer	CCA CAA GTG AGC TGA AGA AGA
**dCINs>MNs:**	
*Ox2r*	
–Forward primer	TAG CCA ATA AGA CCA CCC TCT
–Reverse primer	TGT ACG TCA CCA GAA AGA AGC
*Viaat*	
–Forward primer	TCG TAT GTG GCC ATA GCT AAC
–Reverse primer	GAT ACA CGT CAT CAC CAG CTC
**Housekeeping genes:**	
*Gap-dh*	
–Forward primer	TGG GTG TGA ACC ACG AGA AAT A
–Reverse primer	GCT AAG CAG TTG GTG GTG CAG
*Beta-actin*	
–Forward primer	TCG TAC ACT GGC ATT GTG AT
–Reverse primer	CGA AGT CTA GGG CAA CAT AGC A

The 2^(−ΔΔCT)^ method [100] was used to quantify the difference of mRNA expression of each gene with respect to a housekeeping gene (ΔC_T_) between sample and control (ΔΔC_T_). The analysis was done in R (http://www.r-project.org) on data exported from the ABI Prism® 7000 SDS software (Applied Biosystems).

### Minimizing contamination from surrounding cells

We kept the thickness of the spinal cord sections for LMD at 10 µm to minimize contamination from surrounding cells as described in [5]. Possible contamination from surrounding cells after LMD presumably average out in the analysis over the seven to eight microarray replicas, as the significance analysis of DE genes only detects transcripts that are consistently DE between the experimental groups. We do not expect such minimal and random contamination to produce consistent signals across samples that could produce false positive DE genes. In worst case scenario such a small contamination will introduce noise to the “true” cell-specific expression values, which could prevent detection of differential expression for low expressed genes.

### Microarray probe annotation

It has previously been noted that some old probe sets remain annotated in the Affymetrix chip description files (CDFs) even though analysis on updated genome and transcriptome sequence databases show clear miss matches (MM), i.e. unspecific alignments [101], [102]. These erroneous probe sets should be excluded from the analysis and masked, for instance by altering the CDF files themselves as suggested in [101]. We relied on the Ensembl [103] transcriptome database to filter out obsolete probe sets. The Ensembl transcriptome database only includes Affymetrix probe sets that match the updated transcript sequences. By linking to the Ensembl transcriptome database and retrieving the annotated probe sets for the RN_U34 chip we in essence filter out genes by excluding probe sets not included in this updated list. For the RN_U34 chip this procedure reduced the probe sets with 272 transcripts, making it a total of 1050 not 1322. That means that almost 21% of the probe sets on the RN_U34 chip were obsolete.

The biomaRt bioconductor package (http://bioconductor.org) was used to retrieve updated annotations for the RN_U34 probe sets from the Ensembl transcriptome database.

### Background compensation

Three methods were tested to reduce background noise: Global, MAS (MAS 5.0 software; Affymetrix) and RMA convolution [80]. The Global background compensation is a simple method to minimize the array-wise background level by subtracting the array PM intensity minimum from all PM values, i.e. resetting each array intensity distribution to start at zero prior to normalization. Both MAS and RMA background compensation are described elsewhere [80], [104].

### Normalization

Two widely accepted and popular methods for microarray normalization, QPN and qspline [22], [79], operate on array distributions through nonlinear transformations of the distribution quantiles. These methods assume that the gene expression distributions of different cell types are identical, i.e. any change in the expression of individual genes is balanced such that the overall distribution is preserved. Taken together this implies that any deviation among the measured probe intensity distributions is due to experimental errors and normalization can be carried out to equal these distributions. In order to actually test the hypothesis that all gene expression distributions are identical, we developed a linear scaling method to inspect for similarity among probe intensity distributions. In line with the strategy of the QPN and qspline methods we consider the intensity distributions and hence re-order the data into quantiles, which plotted against a reference distribution produces monotonically increasing curves to which a linear regression can be performed with good results. We thus combine a nonlinear transformation of sorting the data according to rank with a linear fit to the transformed data. The parameters of the fit are then used to rescale the data, in essence making the normalization itself a linear transformation. In this analysis we only use PM values excluding the MM values and make use of probe level normalization in accordance with previous discussions [80], [105], [106]. In particular, the procedure is a linear transformation that maps each array's PM intensity distribution, *I_j_*, onto the average PM distribution of all arrays, *I_ref_*. We refer to this procedure as QLT. For the average reference distribution the *q*-th quantile is calculated as follows:
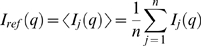
(eq 1)For a total of *m* probes, the mapping between quantiles and ranks, *r*, are given by: *q = r/m|r* = {1, 2, … , *m*}. In short, we therefore sorted the probe intensities of each of the *n* microarrays according to rank *r*, aligned these vectors of ordered intensity values into columns of a matrix and averaged each row to give the average quantiles for the reference distribution (calculated as the QPN procedure). The scaling parameters for each array were obtained from a linear regression between quantiles, *q*, of the average distribution, *I_ref_*, and the PM intensity distribution, *I_j_*, for array *j*:
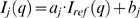
(eq 2)Thus *I_j_ (q)* is a vector of ordered intensity values for array *j*, making this a regression to pair-wise equally ranked intensities. In particular we solve:
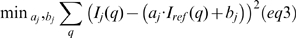
(eq 3)It should be noted here that, as the distributions do deviate at the tails (see Figure 2C), the linear fit to quantiles was carried out on a reduced range: from the 0th to 90th quantile. The variation at the tails only accounts for a small percentage of the data, the majority of which correlate nicely with the average distribution. The range of the linear fit is not fixed and can be adjusted according to the data set under consideration. The parameters, *a_j_* and *b_j_*, from the linear regression are used to rescale array *j:*

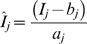
(eq4)
*I* ^*_j_* is the normalized intensity distribution. Under the assumption of equal expression distributions and linear experimental artifacts, this linear scaling should suffice to make all distributions equal irrespective of the nature of these distributions. That is, if two distributions belonging to the same family and therefore only deviate in their distribution parameters, a qq-plot between these will show a linear relationship that deviates from the identity line according to the difference in the distribution parameters.

### Expression summary measures

Li-Wong model based index was used as probe set summary measure [107]. RMA summaries [108] were also tested for comparison. After expression summary calculation all gene expression profiles were minimized by subtraction of the universal minimum of all expression values. This procedure increases ratios of gene expression between samples. Both summary measures were calculated using the affy package from Bioconductor (http://bioconductor.org). The Li-Wong expression summaries for each of the 22 microarrays were submitted to the National Center for Biotechnology Information (NCBI), Gene Expression Omnibus (GEO; http://www.ncbi.nlm.nih.gov/geo/), together with the raw CEL files under accession number GSE9439.

### Quality control

Un-supervised hierarchical clustering with centered Pearson correlation distance measure and principal components analysis were used for the final quality assessment of normalized gene expression summaries. Cluster, pls and amap Bioconductor packages (http://bioconductor.org) were used to calculate and plot the dendogram and principal components.

### Differentially expressed genes

We used a conglomerate classifier based on three different regularized t-test procedures to identify DE genes: Cyber-T [19], limma [18], [109] and SAM [20]. The regularized t-test calculation of either method is implemented for all *n* genes on the microarray. To evaluate the likelihood of DE we use FDR that has been proposed as a good strategy to handle the increasing family wise error rate of multiple testing [23], [110], [111]. For SAM the moderated t-statistic (d score) does not have an associated distribution theory so estimates of reliability are based on empirical FDRs calculated from balanced permutations of samples [20]. Cyber-T and limma on the other hand produce a p-value for each gene, which is used to calculate FDRs. We use two different strategies to calculate the FDR: 1) a mixture model approach as described by Allison *et al* [112] and 2) a step-up FDR controlling procedure suggested by Benjamini and Hochberg [23]. The mixed model approach calculates the expected FDR for a given p-value based on a fit of beta distributions to the histogram of p-values; the higher order beta functions represent the likelihood of true positive and the “null” beta function the likelihood of false positive, which combined can be used to calculate the FDR. Dependence structures in the data manifested as peaks elsewhere in the histogram violates the general assumptions of the model [112]. The other approach, BH FDR, adjusts the p-values by multiplying each p-value with a factor determined by its rank (p-values sorted ascending), *r_i_*, and number of genes, *n*. For gene *i* the adjusted p-value is given by p.adj_i_ = p.raw_i_ * (n/r_i_). To control the FDR we select a FDR = *ϕ* and reject all *p.adj_i_<ϕ* such that *ϕ*% of the rejected null hypothesis' are expected to be false positive. For each test all the genes were ranked according to their test statistic and for each gene the average rank of all three methods were used as a conglomerate classifier of DE.

Calculation of the FDRs for the mixed model approach was carried out with scripts modified from the Cyber-T source code. The BH FDR was implemented by the multtest Bioconductor package in R. Limma and SAM were implemented using the standard Bioconductor packages limma and samr respectively.

### Ratios

For both Affymetrix gene expression summaries and real time RT-PCR data the average ratio of gene expression between the two experimental groups, 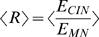, was calculated as the geometric mean of all ratios, *R_i_*, for each gene:
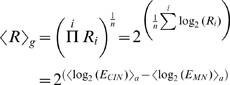
(eq 5)where subscripts *g* and *a* on angled brackets indicate geometric and arithmetic mean respectively and *E* represent expression values. For the average log_2_ transformed ratios, *M*, (as plotted in Figures 4 A-D) we have:
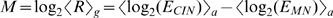
(eq 6)The average log_2_ transformed expression A of any given gene, as used in Figure 4, is given by:
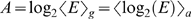
(eq 7)


### Software

All analysis was done using R (http://www.r-project.org) and Bioconductor (http://bioconductor.org), where each R package used is specified in the relevant method section. Cyber-T source code was obtained from the website http://visitor.ics.uci.edu/genex/cybert/. Separate R scripts for QLT normalization, Global background compensation and real time RT-PCR analysis were developed and can be obtained from authors on request.

## Supporting Information

Figure S1 RMA statistics. RMA statistics normalized as Li-Wong (Global bg+QLP+QPN+universal bg). A. Dendogram based on hierarchical clustering of correlation distance between individual RMA expression profiles. B. First two principal components of RMA expression profiles. Symbols: 1 (black) = dCINs, 2 (red) = MIX, 3 (green) = MNs. C. Mixed model fit to Cyber-T p-values, one beta distribution (red): (λ_0_, λ_1_, r, s) = (0.866, 0.134, 0.261, 1.164). D. Mixed model fit to limma p-values, one beta distribution (red): (λ_0_, λ_1_, r, s) = (0.823, 0.177, 0.328, 1.446). Fits to include two or three beta-functions gave similar curves for both limma and Cyber-T. E. Log transformed p-values versus the FDR for limma (black) and Cyber-T (red) for two different measures of FDR. Plot symbols: +represents mixture model FDR based on the fit to one beta distribution parameters from C and D and closed circles are BH FDR. Both methods are indistinguishable at low FDR but deviate slightly at higher FDR. F. Empirical FDR from SAM as a function of its t-statistic (d-score). Horizontal dashed line in E and F correspond to a 10% FDR cutoff.
(2.46 MB TIF)
