# Supplementary figures and images for: Gene Expression Profiling of Two Distinct Neuronal Populations in the Rodent Spinal Cord
Source: PLoS One. 2008 Oct 15;3(10):e3415. doi: 10.1371/journal.pone.0003415 (PMC2566599; doi:10.1371/journal.pone.0003415)

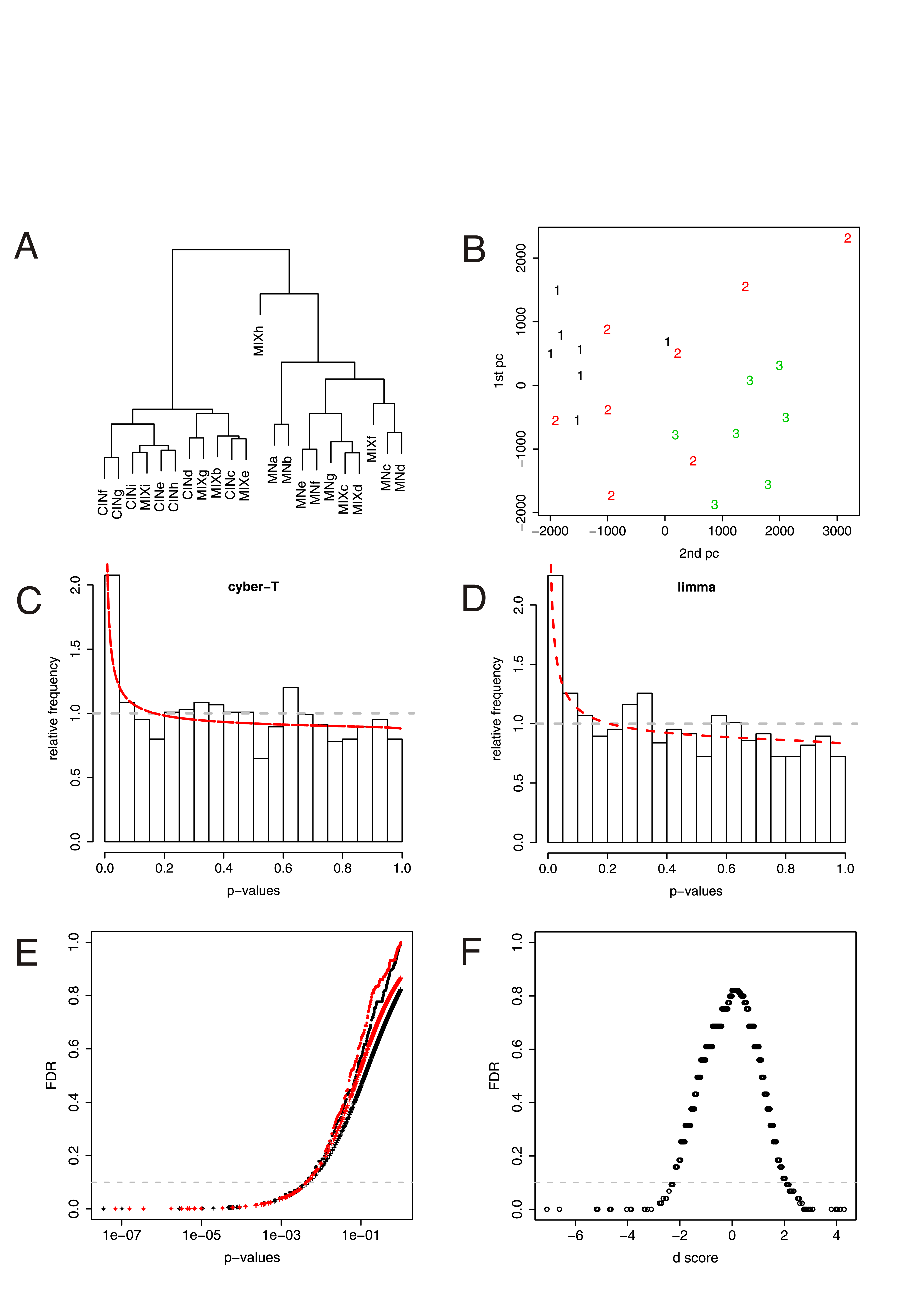

Supplement: Figure S1 — RMA statistics. RMA statistics normalized as Li-Wong (Global bg+QLP+QPN+universal bg). A. Dendogram based on hierarchical clustering of correlation distance between individual RMA expression profiles. B. First two principal components of RMA expression profiles. Symbols: 1 (black) = dCINs, 2 (red) = MIX, 3 (green) = MNs. C. Mixed model fit to Cyber-T p-values, one beta distribution (red): (λ0, λ1, r, s) = (0.866, 0.134, 0.261, 1.164). D. Mixed model fit to limma p-values, one beta distribution (red): (λ0, λ1, r, s) = (0.823, 0.177, 0.328, 1.446). Fits to include two or three beta-functions gave similar curves for both limma and Cyber-T. E. Log transformed p-values versus the FDR for limma (black) and Cyber-T (red) for two different measures of FDR. Plot symbols: +represents mixture model FDR based on the fit to one beta distribution parameters from C and D and closed circles are BH FDR. Both methods are indistinguishable at low FDR but deviate slightly at higher FDR. F. Empirical FDR from SAM as a function of its t-statistic (d-score). Horizontal dashed line in E and F correspond to a 10% FDR cutoff. (2.46 MB TIF) [file pone.0003415.s001.tif]
